# Ferroelectric Switching in Trilayer Al_2_O_3_/HfZrO_x_/Al_2_O_3_ Structure

**DOI:** 10.3390/mi11100910

**Published:** 2020-09-30

**Authors:** Solyee Im, Seung-Youl Kang, Yeriaron Kim, Jeong Hun Kim, Jong-Pil Im, Sung-Min Yoon, Seung Eon Moon, Jiyong Woo

**Affiliations:** 1 ICT Creative Research Laboratory, Electronics and Telecommunications Research Institute, Daejeon 34129, Korea; solyeeim@etri.re.kr (S.I.); kang2476@etri.re.kr (S.-Y.K.); yrar.kim@etri.re.kr (Y.K.); jeonghun@etri.re.kr (J.H.K.); jpim@etri.re.kr (J.-P.I.); semoon@etri.re.kr (S.E.M.); 2 Department of Advanced Materials Engineering for Information and Electronics, Kyung Hee University, Yongin, Gyeonggi 17104, Korea; sungmin@khu.ac.kr; 3 School of Electronics Engineering, Kyungpook National University, Daegu 41566, Korea

**Keywords:** ferroelectric switching, HfZrO_x_, trilayer structure

## Abstract

Since ferroelectricity has been observed in simple binary oxide material systems, it has attracted great interest in semiconductor research fields such as advanced logic transistors, non-volatile memories, and neuromorphic devices. The location in which the ferroelectric devices are implemented depends on the specific application, so the process constraints required for device fabrication may be different. In this study, we investigate the ferroelectric characteristics of Zr doped HfO_2_ layers treated at high temperatures. A single HfZrO_x_ layer deposited by sputtering exhibits polarization switching after annealing at a temperature of 850 °C. However, the achieved ferroelectric properties are vulnerable to voltage stress and higher annealing temperature, resulting in switching instability. Therefore, we introduce an ultrathin 1-nm-thick Al_2_O_3_ layer at both interfaces of the HfZrO_x_. The trilayer Al_2_O_3_/HfZrO_x_/Al_2_O_3_ structure allows switching parameters such as remnant and saturation polarizations to be immune to sweeping voltage and pulse cycling. Our results reveal that the trilayer not only makes the ferroelectric phase involved in the switching free from pinning, but also preserves the phase even at high annealing temperature. Simultaneously, the ferroelectric switching can be improved by preventing leakage charge.

## 1. Introduction

Ferroelectricity based on spontaneous polarization of dipoles in complex ternary oxides (e.g., BaTiO_3_) has been widely used in various electronic and sensor applications [1]. New discoveries of doped HfO_2_ materials suitable for complementary metal oxide semiconductor (CMOS) processes have led to the revival of ferroelectricity in the semiconductor field [2,3,4,5,6,7]. Unlike the ternary ferroelectric materials, which require a certain thickness (~100 nm) to realize the polarization switching, the ferroelectricity can be achieved in extremely thin HfO_2_ layer of less than 20 nm, overcoming integration challenges in the CMOS process. Interestingly, the polarization characteristics were observed in aggressively scaled one-nanometer-thick HfO_2_ films [8]. The attractive ferroelectric properties have been thus widely exploited to improve the sub-threshold slope in the logic transistor due to their negative capacitance [9]. Since the two stable polarization states driven by ferroelectric domains aligned by upward or downward dipoles that can be switched to each other by an electric field are shown, non-volatile memory behavior with binary information has been demonstrated. Successful memory operations have been reported experimentally in dynamic RAM [10] and even 3D vertical stacked NAND architecture [11,12]. In addition, many studies have been attempted to precisely control the number of rotated ferroelectric domains to achieve intermediate polarization states for neuromorphic analog synaptic devices [13,14]. 

To date, optimal dopants for the HfO_2_ material have been studied through material screening by examining the microstructure relevant to the crystallography of the HfO_2_ [15]. Among the dopants such as Si and Al [16,17,18], Zr, which is almost similar to the physical properties of the Hf element, has been representatively used. It has been believed that the observation of the ferroelectricity in hafnia films were contributed by a formation of a non-centrosymmetric Pca2_1_ orthorhombic phase [19,20]. The ferroelectricity was also observed in the undoped HfO_2_ film [21], but incorporating the optimized dopants seems to make the phase transformation more effective and easier. 

In addition to the physical investigations to identify root causes [22,23,24], advanced fabrication processes should be explored and developed. The ferroelectric layers have typically been grown so far with the atomic layer deposition (ALD) technique, which allows sophisticated composition control. However, as an alternative, the sputtering process to deposit the doped HfO_2_ not only allows the polarization switching to be observed, but also enables a cost-effective process that significantly reduces the deposition time compared to the ALD [25,26,27]. In general, the ferroelectricity is shown in the HfZrO_x_ (HZO) materials after annealing at high temperatures that drive phase transformation into the orthorhombic crystalline phase. Note that the fabrication constraints of the ferroelectric devices must be considered differently for each application. When the doped HfO_2_ is used for the memory fabricated on top of the drain of the transistor or stacked vertically in three dimensions, the annealing temperature should be limited as low as possible to avoid malfunction of the metal electrode. Considering the back-end-of-line process, utilizing pressure, which is an alternative driving force, has been considered to demonstrate ferroelectricity at relatively low annealing temperatures [27,28,29]. At a given temperature, the increased pressure can lead to transformation of the crystal structure. On the other hand, the HfO_2_ material can be implemented as a gate dielectric during the front-end-of-line process, where high temperature annealing is performed to activate source and drain of the transistor [30]. In the latter case, since the thermal budget can be negligible, it is more important to ensure immunity to the high temperature. 

Therefore, in this work, we introduced a thin Al_2_O_3_ (AlO) layer to the HZO-based ferroelectric device systems exhibiting a trilayer AlO/HZO/AlO structure for reliable polarization operations. Compared to the properties obtained from the single HZO, we investigated what ferroelectric behaviors were affected by introducing the AlO layers. 

## 2. Experiments 

A 17-nm-thick HZO was deposited on TiN metal serving as a bottom electrode (BE) by RF sputtering from a single stoichiometric Hf_0.5_Zr_0.5_O_2_ target with an Ar plasma at room temperature. After a Pt was deposited by an electron beam evaporator as a top electrode (TE), annealing was performed through rapid thermal annealing at a temperature of 850 °C. The high temperature was raised up for 20 s, and the target temperature was maintained for the next 50 s during annealing. Note that the specific annealing conditions mentioned above constituted the optimum process to demonstrate the ferroelectricity of the sputtered HZO in our preliminary study. The detailed experimental results were discussed in [31]. For the trilayer system, an ultrathin 1-nm-thick AlO layer grown by ALD using a trimethylaluminum precursor was introduced at both the BE and TE interfaces. The ferroelectric devices were characterized by a Keithley 4200 with pulse measurement unit modules. 

## 3. Results and Discussion

**Figure 1:**
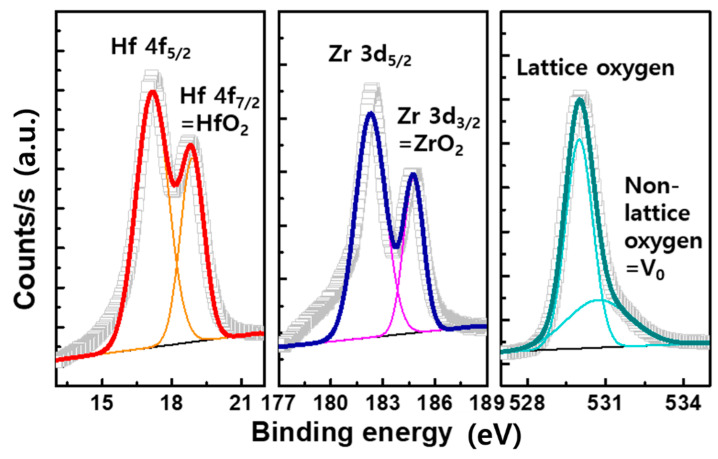
The XPS of the as-deposited 17-nm-thick HZO layer.

**Figure 2:**
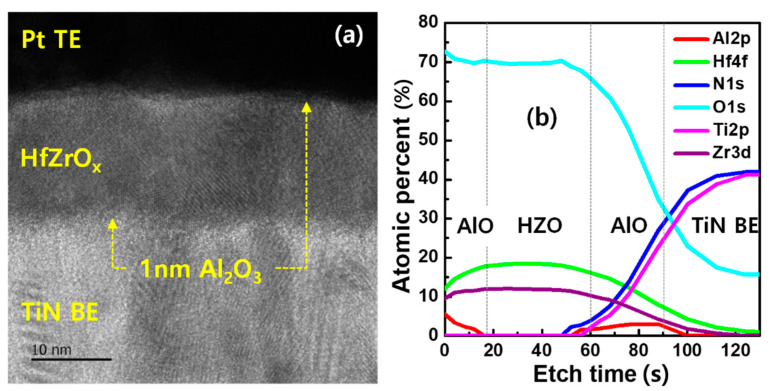
(**a**) Cross-sectional TEM image of the trilayer AlO/HZO/AlO structure. The crystallized phases were shown in the HZO. (**b**) XPS depth profile of the trilayer. The Al peaks were detected at the both interfaces.

First, the physical properties of as-deposited 17-nm-thick HZO layer were analyzed. As shown in Figure 1, atomic percentages of Hf and Zr of 18 and 13 were detected via X-ray photoelectron spectroscopy, respectively. Figure 2a shows a transmission electron microscopic (TEM) image of the trilayer structure. Although the TEM analysis clearly showed crystallized HZO, it was indeed difficult to distinguish the extremely thin AlO layers at both interfaces. Instead, X-ray photoelectron spectroscopy (XPS) depth profiling indicated the presence of the AlO layers. As shown in Figure 2b, an Al 2p peak at the TE surface was increased. The peak was also observed when the Ti 2p peak representing the BE began to be detected, meaning the HZO was sandwiched by the AlO layers.

**Figure 3:**
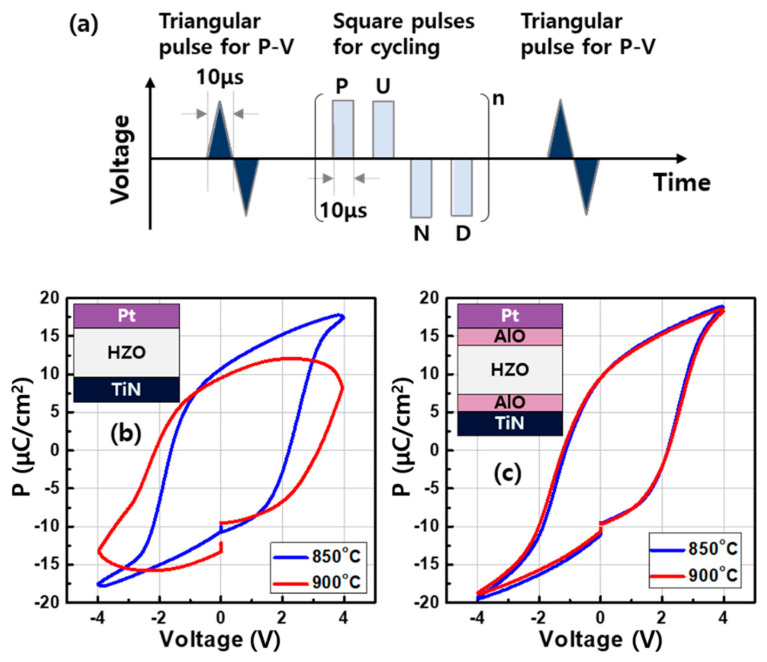
(**a**) The schematic illustration of each pulse for the P–V test and PUND cycling. The P–V curves after 10^3^ PUND cycles of the (**b**) single layer HZO and (**c**) trilayer AlO/HZO/AlO. The devices were annealed via RTA at temperatures of 850 and 900 °C.

The devices were evaluated by applying a triangular pulse with a width of 10 μs corresponding to 100 kHz, as shown in Figure 3a. For a wake-up, 10^3^ cycles of positive-up-negative-down (PUND) pulses with the same width of 10 μs were used. The subsequent square pulses were used to wake up the ferroelectric domains in the HZO and evaluate the fatigue behavior. The pulse set consisting of two consecutive positive pulses followed by two negative pulses called positive-up-negative-down technique was introduced to identify the charges solely induced by polarization [19]. Generally, charges were accumulated in the HZO capacitor not only by polarized dipoles but also by unwanted leakage instantly. By measuring the charges twice at the same state, the response from the leakage could be distinguished and excluded. 

**Figure 4:**
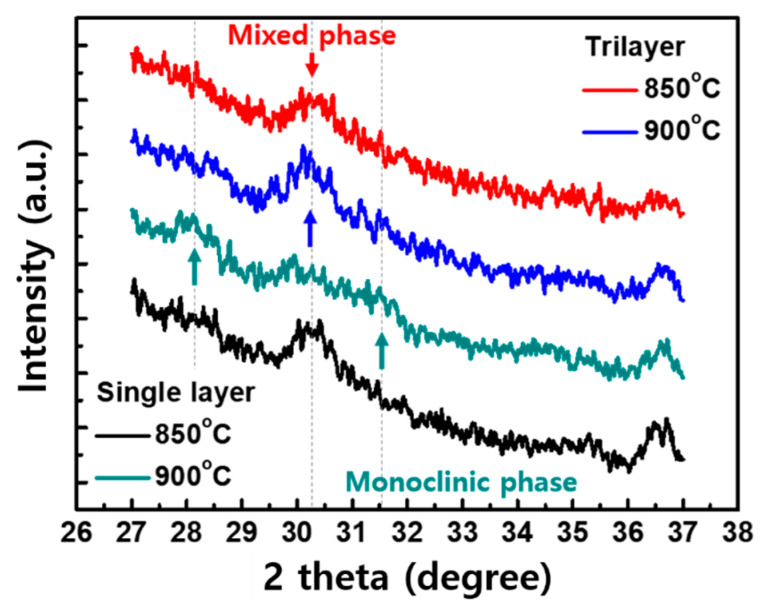
Highest peak intensity at 2 theta of around 30° was shown in the trilayer, whereas the peak was decreased in the single layer annealed at 900 °C.

Figure 3b,c shows the polarization–voltage (P–V) curves of the devices. The pristine single layer HZO was woken-up by the PUND pulses with 2 V to prevent breakdown (Figure 3b). As the applied voltage was increased to +4 V, the polarization was rapidly transited at about +2 V, indicating a coercive voltage (V_c_). A remnant polarization (P_r_) at 0 V greater than 10 μC/cm^2^ was obtained due to spontaneously polarized dipoles in the HZO. When the single layer HZO was annealed at a slightly higher temperature of 900 °C, the P–V curve became rounded at larger voltages. On the other hand, the trilayer system showed the similar P_r_ compared to the single layer and was operated successfully without the breakdown by PUND pulses with a larger voltage of 4 V, as shown in Figure 3c. In addition, stable ferroelectric switching was achieved, even though the trilayer was annealed at 900 °C. These results suggest that orthorhombic phases responsible for the ferroelectricity are well preserved in the trilayer systems. As shown in Figure 4, our result showed that a noticeable peak intensity through X-ray diffraction (XRD) analysis was observed at 2 theta of about 30, indicating the mixed phase including orthorhombic, cubic, and tetragonal phases, when the polarization switching in the sputtered HZO was realized. However, the peak became lower as the annealing temperature was slightly increased. The increased other peaks adjacent the 2 theta of about 28 and 31.5 means that the monoclinic phase seemed to be transformed. It resulted in the degraded polarization behavior in the P–V trace. On the other hand, even at the high annealing temperature of 900 °C, the important mixed phase was still dominant in the trilayer, as shown in Figure 4.

**Figure 5:**
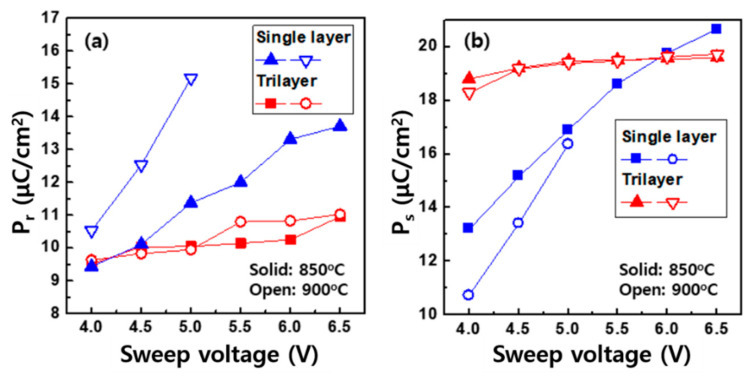
(**a**) P_r_ and (**b**) P_s_ measured at 0 V and 4 V, respectively, as a function of the sweep voltages. The trilayer devices exhibited stable switching parameters over the entire voltage range.

At the given wake-up cycle of 10^3^, the P_r_ in the trilayer remained almost constant when sweeping the voltages larger than 6 V, no matter what annealing temperature was used, as shown in Figure 5a, whereas the P_r_ in the single layer was proportionally increased as a function of the voltage amplitude. Similar trends were observed not only in the P_r_ but also in the saturation polarization (P_s_), defined as the polarization measured at 4 V, as shown in Figure 5b. The change in these parameters is related to whether the ferroelectric phases in the HZO participating in the switching are easily transformed. Basically, the deposited HZO layer is crystallized through the annealing to form the orthorhombic phases. During the phase transition, several phases are pinned by defects, resulting in the non-ferroelectric state [23]. The 10^3^ PUND pulses were thus used to wake-up the inactivated phases. The enlarged voltage application also eliminated the defects away from the phases. These caused more phases to be involved in the ferroelectric switching. As shown in Figure 5, the parameters such as P_r_ and P_s_ in the trilayer were less sensitively affected by the electrical stimulation. This means that more active ferroelectric phases could be formed, which is in good agreement with the physical results obtained from the XRD. Meanwhile, the single layer HZO annealed at 900 °C seemed to be more vulnerable to the voltage stress, worsening the switching stability. Although the transition of the polarization at the V_c_ in the HZO occurred, the P–V curve was incompletely saturated in the large voltage range (Figure 3b). This was because the charges induced by the electric field were added to the P_r_, which was derived by the spontaneously polarized charges. The trilayer system was able to minimize the leakage charge, resulting in the stable parameters. 

**Figure 6:**
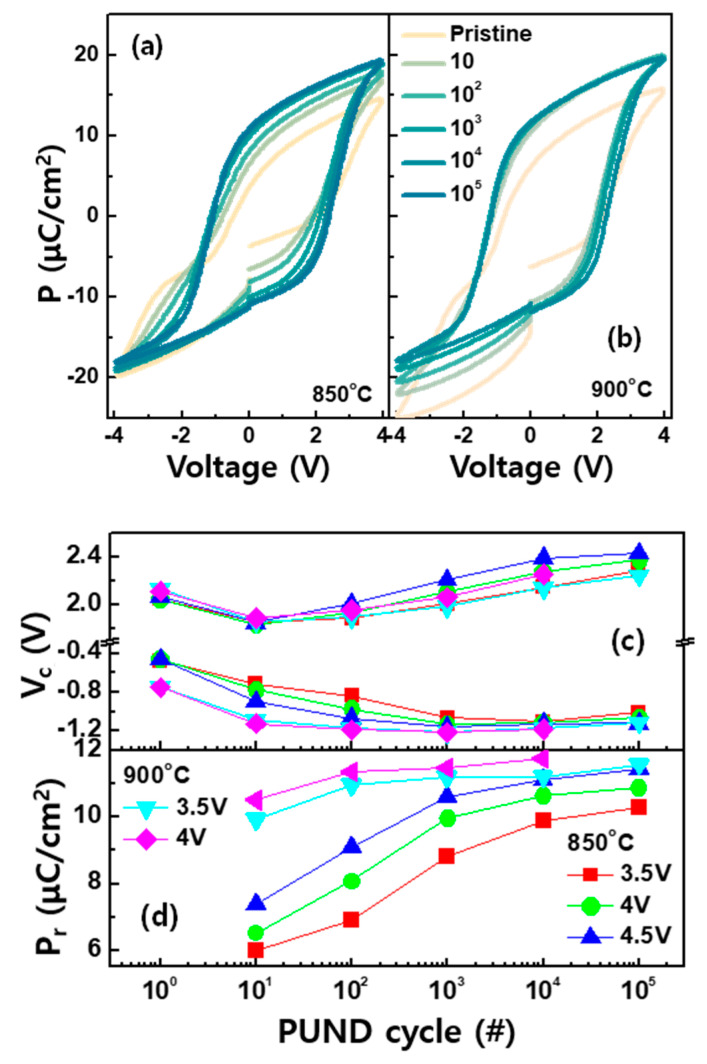
The P–V curves of the trilayer annealed at (**a**) 850 °C and (**b**) 900 °C. The extracted (**c**) ±V_c_ and (**d**) P_r_ as a function of the voltage amplitude of the PUND cycles.

Next, we examined the P–V curves in the trilayer systems as a function of the PUND cycles to understand the impact of the annealing temperature. Figure 6a,b shows that the P–V traces after the PUND cycles with ±4 V were applied to the pristine state. Both trilayer devices initially exhibited pinched hysteresis, meaning weak antiferroelectricity [32]. As the PUND cycles were addressed to the device, the property was converted to the ferroelectricity. Note that the switching parameters such as P_r_ and ±V_c_ were more gradually changed in the device annealed at 850 °C. To achieve the P_r_ close to 10 μC/cm^2^, 10^3^ PUND cycles were needed (Figure 6a,c). However, for the device annealed at 900 °C, only 10 cycles were required (Figure 6b,d). Unlike the single layer, where unwanted phase transformation occurred at high temperature, no distinct structural modification was observed in the trilayer (Figure 4). Rather, the high temperature seemed to be advantageous, by annihilating the pinned defects from the active ferroelectric phase. Thus, only a small amount of non-ferroelectric phases was needed to be activated, resulting in P_r_ quickly reaching a stable value. For the same reasons mentioned earlier, the larger PUND voltage at a given cycle accelerated the transition. 

**Figure 7:**
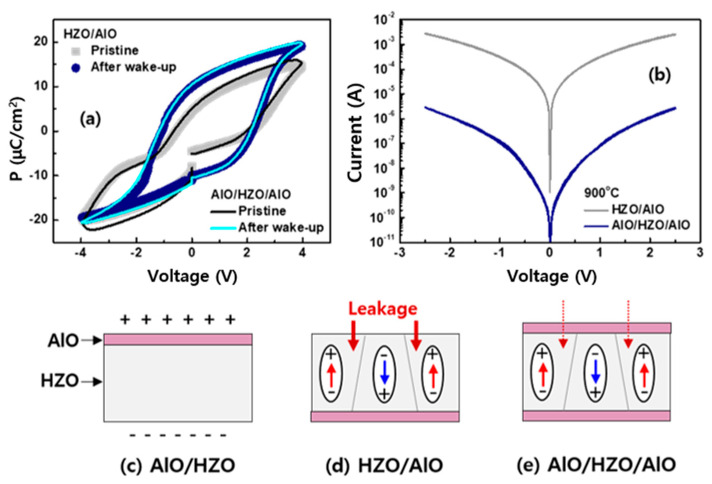
(**a**) The P–V curves of the device systems annealed at 850 °C. (**b**) I–V traces of both device systems annealed at 900 °C. The dielectric and spontaneous polarizations were shown in (**c**) AlO/HZO and (**d**) HZO/AlO structures, respectively. (**e**) Stable ferroelectric switching was observed in the trilayer systems annealed at the higher temperature of 900 °C.

We then investigated bilayer systems annealed at 850 °C to identify the AlO layer’s role. The AlO located on top of the HZO showed a dielectric polarization temporally induced by the applied field (not shown here). When the AlO layer was inserted only between the HZO and BE, the ferroelectric switching began to be observed in Figure 7a. However, increased annealing temperature to 900 °C caused the breakdown of the bilayer HZO/AlO (from top to bottom), as we observed in the single HZO layer. The current–voltage (I–V) characteristic showed that the failure was related to the leaky HZO/AlO bilayer due to the high temperature annealing, as shown in Figure 7b. The capped AlO layer was expected to serve as a barrier to prevent additional leakage charges in the trilayer systems, as shown in Figure 7c–e. In addition, the inserted AlO layer could prevent unwanted chemical reactions between the TiN electrode and HZO during annealing [33], creating a TiO_x_ interfacial layer with oxygen vacancies strongly affecting the ferroelectric properties of the HZO. 

Similar to this work, bilayer systems with HZO/AlO ferroelectric/dielectric structures were recently reported [34,35]. Unlike the dielectric, ferroelectric (or antiferroelectric)-related layers such as ZrO_2_ serving as a seed layer were introduced to promote the orthorhombic phase in the doped HfO_2_ film [36,37]. Additional thin films have begun to be introduced rather than single HfO_2_, but their role and impacts on the polarization and reliability characteristics have not been elucidated. Further research to clarify these aspects is currently underway based on the fabricated multilayer ferroelectric devices. Here, we focused on the polarization behavior in a simple capacitor structure that provided a good understanding of intrinsic ferroelectric properties. When the ferroelectric switching layer was integrated into the transistor structure called ferroelectric FET (FeFET), the achieved polarization was somewhat degraded, resulting in a smaller on/off ratio for non-volatile memory applications. Thus, it remains a challenge to identify how the switching parameters such as the P_r_ and V_c_ of the ferroelectric layer are linked to reliability characteristics such as data retention and cycling endurance in the FeFET. 

## 4. Conclusions

The recent discovery of the ferroelectricity in the doped HfO_2_ layer has led to great interest for next-generation non-volatile memory and neuromorphic applications. It is worth noting that the fabrication process to realize the polarization in the layer needs to be appropriately designed depending on the device configuration aimed at the target application. In this regard, we investigated the ferroelectric characteristics of the sputtered HZO film under higher temperature applications. The ferroelectric properties of the single layer HZO were vulnerable to external environments such as higher annealing temperature, causing the unwanted phase transition. The sweeping voltages accompanied by electrical stress also made the switching parameters such as P_r_ and P_s_ be disturbed by the leakage components. On the other hand, the trilayer AlO/HZO/AlO systems allowed the ferroelectric phase to be preserved even at the high annealing temperature. Rather, the temperature led the non-ferroelectric phases to be released quickly from the pinning, requiring only 10 PUND cycles for stabilization.
